# Gut microbiota is associated with differential metabolic characteristics: A study on a defined cohort of Africans and Chinese

**DOI:** 10.3389/fendo.2022.942383

**Published:** 2022-09-28

**Authors:** Paul Nizigiyimana, Boya Xu, Lerong Liu, Liping Luo, Tingting Liu, Meng Jiang, Zehao Liu, Changjun Li, Xianghang Luo, Minxiang Lei

**Affiliations:** ^1^ Department of Endocrinology, Endocrinology Research Center, Xiangya Hospital of Central South University, Changsha, China; ^2^ Department of Endocrinology, Haikou Hospital Affiliated to Xiangya School of Medicine, Central South University, Haikou, China

**Keywords:** Chinese, Africans, healthy, gut microbiota, 16S rRNA gene sequencing, bacterial communities

## Abstract

**Objective:**

This study intended to determine the associations between gut microbiota and glucose response in healthy individuals and analyze the connection between the gut microbiome and glucose-metabolism-related parameters.

**Methods:**

Fecal bacterial composition and anthropometric, body composition, body fat distribution, and biochemical measures were analyzed. A 75-g oral glucose tolerance test (OGTT) was given to each participant to investigate changes in glucagon-like peptide 1 (GLP-1), insulin, and glucose. The whole body fat and the regions of interest of local body composition were analyzed using dual-energy X-ray absorptiometry (DEXA), and gut microbiota composition was assessed through variable regions (V3–V4) of the bacterial 16s ribosomal RNA gene using high-throughput sequencing techniques. Spearman correlation analysis was used to evaluate the association between gut microbiota and clinical and metabolic changes.

**Results:**

The number of operational taxonomic units (OTUs) demonstrated a reduction in the diversity and composition of gut microbiota associated with enhanced adiposity, dyslipidemia, insulin resistance, and hyperglycemia. The alpha diversity revealed that microbiota diversity, richness, and composition were higher in the African group and lower in the Chinese group. Principal coordinates analysis (PCoA) plots of beta diversity showed significant variability in gut microbial community structure between the two groups (*p* = 0.0009). LEfSe analysis showed that phylum Bacteroidetes was significantly more abundant in the Chinese group, and this group also harbored members of the order Bacteroidales, family Bacteroidaceae, and genus *Bacteroides*. In contrast, the phylum Verrucomicrobia was significantly more prevalent in the African group (all *p* < 0.05). Concerning species, metastats analysis revealed 8 species in the Chinese group and 18 species in the African group that were significantly abundant. Spearman’s correlation analysis demonstrated that gut microbiota correlated with the factors that related to glucose metabolism.

**Conclusion:**

Our data suggest that there is an interaction between gut microbiota, host physiology, and glucometabolic pathways, and this could contribute to adiposity and pathophysiology of hyperlipidemia, insulin resistance, and hyperglycemia. These findings provide an important basis for determining the relation between the gut microbiota and the pathogenesis of various metabolic disorders.

## 1 Introduction

Obesity and T2DM are the most prevalent metabolic disorders and worldwide major health challenges today. The incidence of T2DM is rising globally (1, 2) with a high increase in Asian countries (1, 3, 4). Plenty of genomic studies have reported connections between gut microbiota and metabolic disorders such as obesity, insulin resistance, and T2DM, and this gives an idea that a causal relationship could exist. The pathogenesis of obesity, insulin resistance, and T2DM in Asians and Africans is very different (5–9). It is unclear whether these pathophysiological differences may be related to differences in the gut microbiota. The gut microbiota is the intestinal microbial community that performs an important role in maintaining the host physiology, sustaining health, and disease pathogenesis. Currently, the gut microbiota is increasingly recognized as an endocrine organ that maintains host energy homeostasis and contributes to host immunity (10, 11). Earlier studies on gut microbiota have suggested that the composition of gut microbiota can contribute to health and is closely associated with metabolic disorders. For example, alteration (dysbiosis) of gut microbiota can lead to a dramatically altered symbiotic relationship between gastrointestinal microbiota (gut bacteria) and the host, which contributes to the development of obesity, metabolic syndrome, T2DM (12, 13), and cardiometabolic disease (12), and non-alcoholic fatty liver disease (12, 14). Moreover, this dysbiosis of the gut microbiota may give rise to a pathophysiological mechanism underlying systemic inflammation in insulin resistance (15, 16). Despite the immense contributions of gut flora in multiple disorders, gut microbiota characteristics in healthy Chinese and Africans are poorly understood. It has been suggested that the gut microbiota can involve in the modulation of host energy metabolism, fat storage, glucose control, and insulin sensitivity by regulating certain factors such as fats, lipids, bile acids, and glucagon-like peptide 1 (GLP-1) that participate in metabolic pathways of glucose metabolism (12, 17, 18). Current studies have highlighted gut microbiota as a new therapeutic target to improve metabolic health (12), although many factors (e.g., diets, lifestyle changes, urbanization, environmental conditions, and genetic factors) have been reported to shape the gut microbiota community (19–25), making it difficult to perform vital functions like nutrition, physiology, metabolism, and immune function. A growing understanding of the risk factors that impact the incidence of metabolic disorders such as obesity, insulin resistance, and diabetes can disseminate advanced knowledge on the pathophysiology of these disorders and can facilitate adopting treatment or prevention strategies and/or measures to delay their occurrence. This study evaluated the associations between gut microbiota and glucose response in healthy individuals and analyzed the connection between gut microbiota and factors related to glucose metabolism.

## 2 Materials and methods

### 2.1 Subject enrollment criteria

This study recruited 27 Han-Chinese and 29 African citizens (university students) living in Changsha, China *via* on-campus advertisements. These African citizens were born in Africa (Burundi, Rwanda, Uganda, and Tanzania) and have no known recent non-African ancestry. Participants were male and female with age 18–35 years (**Table 1**) who were metabolically stable within the last 6 months and had a long stay in China for at least 1 year, with no participation in any clinical trial until the day of the study and had no special food habits. Exclusion criteria included pregnancy, lactation, smoking, and history of chronic metabolic diseases and/or neurological, autoimmune, and gastrointestinal diseases. Subjects taking any medication that interferes with insulin, glucose, and GLP-1 or with treatments affecting gut permeability, motility, or microbiota were also excluded. We confirmed health status through blood pressure measurements and lipid and biochemical profiles (**Table 1**) and by the absence of glucose intolerance (26).

**Table 1:** Anthropometric profiles, body composition and body fat distribution, and biochemical and clinical measurements in Chinese and African subjects.

Characteristic	Chinese (n=27)	Africans (n=29)	*p*-value
Gender:
Male, n (%)	19 (70.4%)	21 (72.4%)	–
Female, n (%)	8 (29.6%)	8 (27.6%)	–
Age (year)	25.07 ± 1.49	26.1 ± 4.24	0.895
Height (cm)	171.63 ± 7.41	173.07 ± 8.15	0.493
Weight (kg)	66.33 ± 9.80	67.45 ± 11.53	0.699
BMI (kg/m^2^)	22.46 ± 2.48	22.53 ± 3.56	0.932
Waist circumference (cm)	76.06 ± 10.71	79.66 ± 9.16	0.182
Hip circumference (cm)	90.66 ± 9.10	95.82 ± 9.26	**0.040**
Waist–hip ratio	0.84 ± 0.07	0.83 ± 0.04	0.621
Arm circumference (cm)	27.72 ± 3.77	27.31 ± 3.43	0.673
Systolic BP (mmHg)	112.74 ± 9.49	112.1 ± 10.43	0.812
Diastolic BP (mmHg)	73.03 ± 8.62	69.76 ± 6.32	0.109
SMI (kg/m^2^)	15.51 ± 2.08	15.17 ± 2.36	0.568
LS-BMD (g/cm^2^)	0.98 ± 0.09	1.01 ± 0.10	0.081
PF-BMD (g/cm^2^)	0.98 ± 0.12	0.99 ± 0.14	0.699
Total body BMD (g/cm^2^)	1.17 ± 0.06	1.15 ± 0.09	0.360
Body fat (%)	26.77 ± 5.57	27.05 ± 8.82	0.885
A/G ratio	1.06 ± 0.19	0.87 ± 0.17	**<0.001**
FMR_trunk-to-limb_	1.08 ± 0.23	0.85 ± 0.17	**<0.0001**
Trunk/leg fat ratio	1.02 ± 0.22	0.87 ± 0.13	**0.003**
Total cholesterol (mmol/L)	4.22 ± 0.70	4.36 ± 0.88	0.222
LDL cholesterol (mmo/L)	2.42 ± 0.66	2.53 ± 0.81	0.549
HDL cholesterol (mmol/L)	1.44 ± 0.28	1.53 ± 0.34	0.275
Triglycerides (mmol/L)	1.03 ± 0.59	0.83 ± 52	**0.039**
Total bile acids (µmol/L)	5.37 ± 5.56	3.24 ± 2.93	**0.049**
Total bilirubin (µmol/L)	14.60 ± 5.32	11.44 ± 5.49	**0.022**
Direct bilirubin (µmol/L)	6.54 ± 2.26	4.88 ± 2.30	**0.009**
Fasting GLP-1 (ng/ml)	0.28 ± 0.33	0.19 ± 0.08	0.652
Fasting insulin (µU/ml)	6.97 ± 2.25	6.56 ± 2.42	0.513
Fasting glucose (mmol/L)	4.58 ± 0.40	4.61 ± 0.46	0.774
Δglucose (30–0 min)	2.68± 1.08	1.94± 1.17	**0.017**
DI_180_	95.83 ± 28.87	115.10 ± 42.87	0.056
Matsuda index	7.08 ± 2.47	8.36 ± 4.10	0.314
HOMA-IR_30min_	20.36 ± 8.66	16.36 ± 10.64	**0.026**

Data are reported as means and standard deviations (X ± SD).

BMI, body mass index; BP, blood pressure; SMI, skeletal muscle mass index; LS-BMD, lumbar spine bone mineral density; PF-BMD, proximal femur bone mineral density; A/G ratio, android/gynoid ratio; FMR_trunk-to-limb_, trunk/limb fat mass ratio; LDL, low-density lipoprotein; HDL, high-density lipoprotein; GLP-1, glucagon-like peptide 1; Δ Glucose (30–0 min), incremental glucose level at 30 min; HOMA-IR_30min_, homeostatic model assessment for insulin resistance at 30 min; DI_180_, a disposition index obtained from the product of AUCins_0–180_/AUCglu_0–180_ ×Matsuda index. The bold for values was just to emphasize the statistical significance.

### 2.2 Anthropometric measurements and biochemical analysis

All subjects were refrained from doing vigorous exercise and underwent overnight fasting of 12 h. Height, weight, blood pressure, and circumferences of arm, waist, and hip were measured, and a standard 75-g oral glucose tolerance test (OGTT) was given to each participant early in the morning at 8 a.m., and venous blood samples were drawn at the time points of 0, 30, 60, 120, and 180 min for the measurements of glucose, insulin, and GLP-1. The samples for the determination of GLP-1 levels were collected in tubes free of aprotinin or DPP-IV. Sample tubes were centrifuged at 1,000×*g* for 15 min at 4°C, and the resulting supernatants were collected and stored at −80°C until analysis of plasma total GLP-1 and insulin. The levels of triglycerides, total cholesterol, high-density lipoprotein (HDL), low-density lipoprotein (LDL), glucose at fasting and glucose during an OGTT, and total bile acids were measured by a Beckman-AU680 automatic biochemistry analyzer with Beckman Coulter kits and Leadman kit, respectively. The hexokinase method was applied for glucose measurements, while automated enzymatic methods were used for lipid profiles and total bile acids. Direct and total bilirubin levels were detected using the diazo method, Azobirubin (Beckman Coulter, USA). Insulin concentrations were detected using chemiluminescent microparticle immunoassay with ARCHITECT kits (DENKA Seiken Co., Ltd., Tokyo, Japan), and the levels of total GLP-1 were measured using ELISA with Elabscience kits (Elabscience Biotechnology Co., Ltd., Wuhan, China).

### 2.3 Body composition assessment and determination of fat distribution

Subjects were asked to empty their bladder and remove any metallic objects before the scan. Subjects were also instructed to breath normally and not talk or move (lie still) during the entire scan for approximately 7 min. Dual-energy X-ray absorptiometry (Hologic QDR 4500A, Hologic Corporation, USA) was used to evaluate fat and bone mineral density (BMD) in the whole body and BMD in the lumbar spine (L1–L4) and proximal femoral. Fat distribution patterns were automatically calculated by performing and executing the analysis (Hologic APEX for Windows software version 5.5.3) according to the operator’s standard analysis protocol.

### 2.4 Gut microbiota analyses

#### 2.4.1 DNA extraction, library preparation, and high-throughput sequencing (16s rRNA gene sequencing)

Fecal samples were collected at baseline, and DNA was extracted using the QIAamp Fast DNA Stool Mini Kit (Qiagen, Hilden, Germany). Based on the preliminary quantitative results of agarose gel electrophoresis, the concentration and purity of sample libraries were assessed by an Invitrogen Qubit 3.0 spectrophotometer (Thermo Fisher Scientific, USA). The quantity and size distribution of DNA fragment libraries and validation of biological replicates were determined using an Agilent 2100 bioanalyzer (Agilent Technologies, USA). The V3–V4 region of the rRNA gene was amplified and then subjected to high-throughput profiling of microbial communities using the MiSeq platform (Illumina, USA). Simply, the 16S rRNA V3–V4 region was amplified using primers 341F (5′-CCTACGGGNGGCWGCAG-3′) and 805R (5′-CCTACGGGNGGCWGCAG-3′), and 250-bp paired-end reads were generated.

#### 2.4.2 Sequencing quality control, data processing, and gut microbial community analysis

The mothur version 1.41.1 was used to generate the reads for further analysis. USEARCH was applied to conduct filtering of the duplicated sequences and chimera removal. The lasting sequences were grouped into operational taxonomic units (OTUs) with a 97% threshold of similarity using the UPARSE and then categorized against the SILVA database.

#### 2.4.3 Calculations and statistical analyses

We determined the presence of insulin resistance by applying the transformed homeostasis model assessment (HOMA-My) (27) for insulin resistance (IR). The transformed HOMA-My indices were obtained using the following formula: Iy (µIU/ml) × Gy (mmol/L)/22.5, where y indicates 30, 60, 120, or 180 min insulin (I) and glucose (G) values from the OGTT. Insulin secretion derived from the OGTT was obtained from the product of the insulin/glucose 0–180 min total areas under the curve ratio and the Matsuda index (AUCins_0–180_/AUCglu_0–180_ × Matsuda index) to assess beta cell function (28) and was expressed as disposition index (DI_180_). The areas under the curves (AUCs) for insulin and glucose were calculated using the trapezium rule (29). The SPSS software (v.18.0.0) was used to perform analyses for baseline anthropometric profiles, body composition and body fat distribution, biochemical and clinical measurements, and data from OGTT. Independent *t*-tests, Mann–Whitney U test, and χ^2^ test were used for parametric, non-parametric, and categorical data to assess differences in measurements between groups, respectively. Alpha diversity was determined based on biodiversity metrics (observed species, Chao1, and Shannon index) to analyze the disparity in the gut microbiota richness and diversity using Wilcoxon tests. Venn diagram was generated using the R package (v1.6.20) and rarefaction and Shannon–Wiener curves were plotted using ggplot2 in R (v3.3.0). Beta diversity was determined based on OTU counts in line with the Bray–Curtis distance metric (30). Principal coordinates analysis (PCoA) was conducted to visualize similarities or differences between data, and permutational multivariate analysis of variance (PERMANOVA) with Adonis was used to evaluate the significant variation in microbial communities between groups. Metastats analysis was performed at multiple taxonomic levels (phylum, class, order, family, genus, and species) to identify differentially abundant taxa between the two groups. Additionally, Benjamin and Hochberg’s false discovery rate (FDR) method was applied to correct and adjust p-values. Linear discriminant analysis (LDA) effect size (LEfSe) with the default alpha value of 0.05 was carried out using the available website “http://huttenhower.sph.harvard.edu/galaxy/root?tool_id=PICRUSt_normalize” to screen taxa that serve mostly as biomarkers between the two groups of participants. Spearman’s rank correlation coefficient was applied to determine the relationships between the sequencing data and other data.

## 3 Results

### 3.1 Study population

The anthropometrical, clinical, biochemical, and body composition characteristics of the participants are given in **Table 1** and **Supplementary Table S1**. We further analyzed these characteristics based on sex-specific classification, and data from this analysis are summarized in **Supplementary Table S2**.

### 3.2 Microbiota profiles in Chinese and African subjects

**Figure 1:**
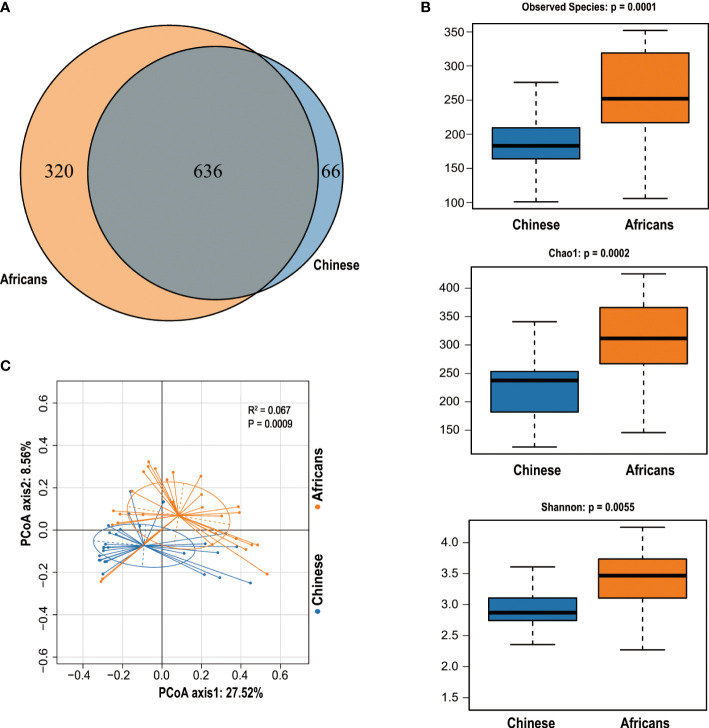
The Venn diagram and community diversity analysis. The Venn diagram depicts the overlapping OTUs between Chinese and Africans **(A)**. The Alpha diversity metrics used to estimate microbial richness and diversity **(B)**. PCoA was computed to display the variability of gut communities among all samples from Chinese and Africans **(C)**. A permutational multivariate analysis of variance (PERMANOVA) with Adonis on Bray–Curtis distances confirmed these differences (R^2^ = 0.067, *p* = 0.0009).

We obtained a total of 4,470,507 reads from 56 samples, with 79,830 reads estimated as an average for each sample. The statistics and quality score of sequencing reads used in this study are detailed in **Supplementary Table S3**. The reads were clustered into 1,022 OTUs based on a similarity score of 97% at the 16S rRNA gene. A Venn diagram demonstrated that 636 OTUs were shared by both groups, whereas 66 OTUs and 320 OTUs were unique for the Chinese and Africans, respectively (**Figure 1A**).

### 3.3 Alpha diversity and beta diversity in Chinese and African subjects

The OTUs evaluation in respect of diversity indices exhibited pronounced variations between groups. The rarefaction curves demonstrated that all samples were detected for OTUs and approached a plateau, which evidences the adequacy of our sequencing data for this study (**Supplementary Figure S1A**), and the Shannon diversity index indicates that the microbial diversity of the gut flora in the African group was the highest and that in the Chinese group was the lowest (**Supplementary Figure S1B**). Indeed, rank-abundance curves indicated that the African group stands out for species abundance, richness, and evenness (**Supplementary Figure S2**). To better understand the distribution and diversity of microbial communities in these two groups, we evaluated the overall community heterogeneity by measuring ecological indices based on Alpha diversity using Wilcoxon tests. The finding revealed that the Alpha diversity decreased significantly in the Chinese group (all *p*<0.05, **Figure 1B**). To determine the dissimilarities in microbial community structures between the Chinese and African group, we calculated beta diversity based on Bray–Curtis distances. The PCoA plot with Bray–Curtis distance matrix revealed that the gut microbiota samples from the African group were clustered separately from the Chinese group, with 27.52% (X-axis) and 8.56% (Y-axis) of the total variance in microbiota composition (Adonis, p = 0.0009, **Figure 1C**).

### 3.4 Composition and abundance of gut microbiota in Chinese and African subjects

**Figure 2:**
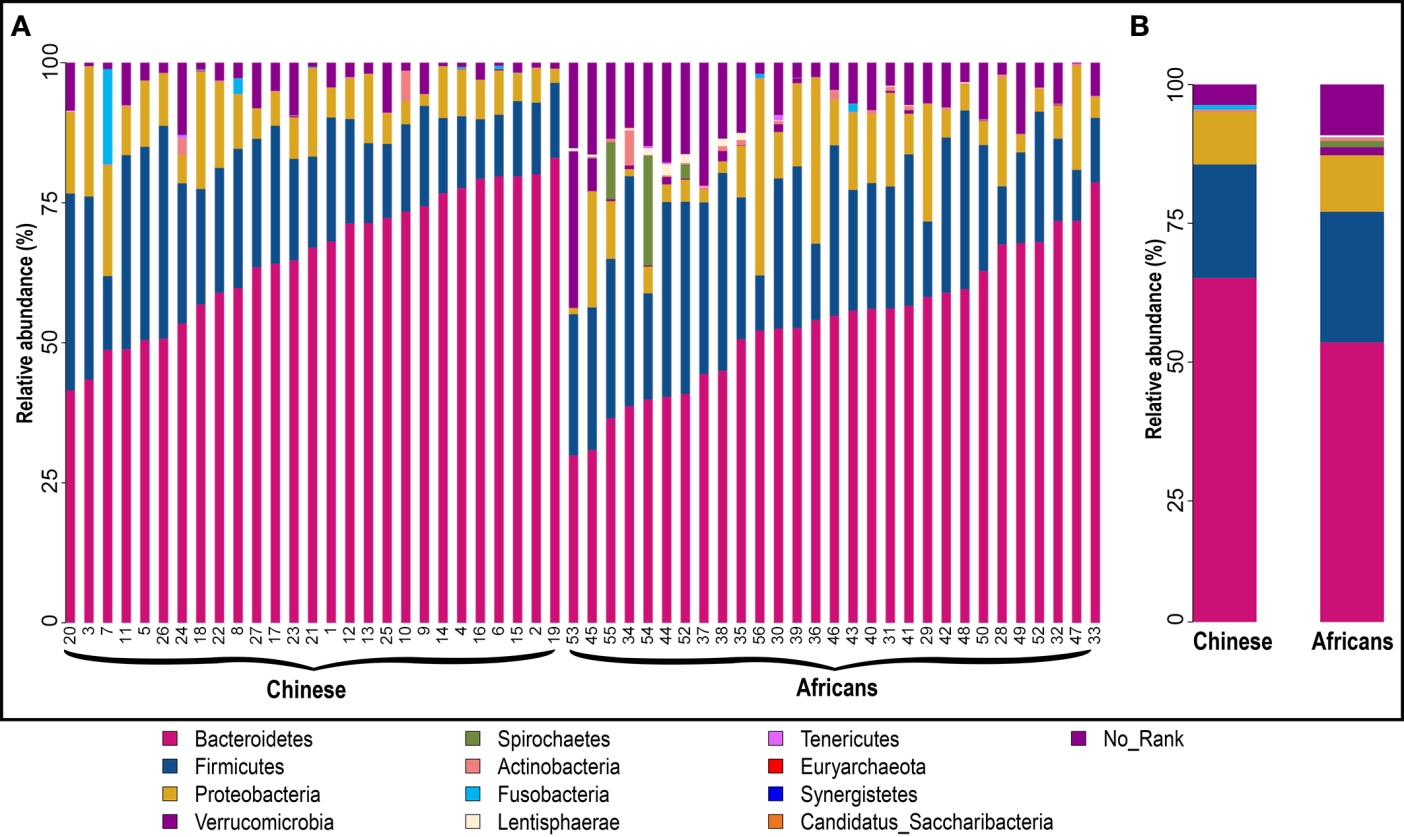
Structural composition of gut microbiota community in the two groups. Microbial composition and their relative abundance in each sample **(A)** and between groups **(B)** at phylum level. The taxa with relative abundance ≥1% are presented. The remaining unmapped taxa are grouped as “No_Rank.” Each bar denotes a single sample or group, and each color represents the relative abundance in percentage for each OTU.

The statistics of the OTUs indicated that they were grouped in 12 phyla (**Figure 2A**). To better understand how the gut microbial community composition differs between the Chinese and African group, we examined which microbial groups were present at multiple taxonomic levels together with their relative abundance. Metastats analysis showed that the taxonomic compositions differed between the two groups. At the phylum level, Bacteroidetes was the most widely represented in both groups, with a relative abundance of 65.14% in the Chinese group and 53.54% in the African group. Firmicutes was the second most widely abundant, accounting for the relative abundance of 20.45% and 23.54%, respectively. The next widely abundant phyla were Proteobacteria, Verrucomicrobia, and Spirochaetes (**Figure 2B** and **Table 2**). At the class level, the Chinese group showed significantly greater relative abundance of Bacteroidia (64.91% vs. 52.71%, *p*<0.001) than the African group. We also observed that the relative abundance of Spirochaetia was absent in Chinese (*p*>0.05). There were no differences in abundances of Clostridia, Negativicutes, Betaproteobacteria, and Gammaproteobacteria (all *p*>0.05, **Table 2**). At the order level, the Chinese group had a significant increase in relative abundance of Bacteroidales (64.91% vs. 52.71%, *p*<0.001) than the African group, whereas the relative abundance of Aeromonadales (0% vs. 1.82%, *p*<0.05) together with Spirochaetales (*p*>0.05) was completely absent in the Chinese group (**Table 2**). On the other hand, the relative abundances of Selenomonadales and Acidaminococcales were predominant in the Chinese group but absent in the African group (*p*>0.05). There were no differences in abundances of Clostridiales, Burkholderiales, and Enterobacterales (all *p*>0.05, **Table 2**). At the family level, the members of the family Oscillospiraceae and Succinivibrionaceae were significantly absent in the Chinese group (all, *p*<0.05, **Table 2**). In addition, the member of the family Spirochaetaceae was completely absent in this group (*p*>0.05). On the other hand, the Chinese group had a significant increase in relative abundance of Bacteroidaceae (43.22% vs. 22.24%, *p*<0.01) than the African group and the member of the family Clostridiaceae was significantly absent in the African group (*p*<0.05, **Table 2**). In addition, members of the family Selenomonadaceae and Acidaminococcaceae were also predominant in the Chinese group but absent in the African group (all *p*>0.05, **Table 2**). There were no differences in abundances of members of the family Porphyromonadaceae, Rikenellaceae, Prevotellaceae, Lachnospiraceae, Ruminococcaceae, Sutterellaceae, and Enterobacteriaceae (all *p*>0.05, **Table 2**). At the genus level, the genera *Parasutterella* (*p*<0.01, **Table 2**) and *Succinivibrio* (*p*<0.05, **Table 2**) were significantly absent in the Chinese group, and also the relative abundance of *Treponema* was completely absent in this group (*p*>0.05). However, the Chinese group had a significant increase in relative abundance of *Bacteroides* (42.83% vs. 22.17%, *p*<0.01, **Table 2**) than the African group. The genera *Megamonas* and *Phascolarctobacterium* were predominant in the Chinese group but absent in the African group (*p*>0.05). There were no differences in abundances of genera *Parabacteroides*, *Alistipes*, *Prevotella*, *Ruminococcus*, *Faecalibacterium*, *Sutterella*, and *Escherichia* (all *p*>0.05, **Table 2**). The number and proportion of unmapped reads (No_Rank) at each taxonomic level are presented in **Supplementary Table S4**.

**Table 2:** Most abundant bacterial taxa in the Africans (n=29) and Chinese (n=27).

Taxon	Annotation	Mean relative abundance		*p*-value	
		Chinese	Africans	*p*-value	FDR
Bacteroidetes	**Phylum**	65.14%	53.54%	**0.000999**	0.0025974
Bacteroidia	Class	64.91%	52.71%	**0.000999**	0.007659
Bacteroidales	Order	64.91%	52.71%	**0.000999**	0.013986
Bacteroidaceae	Family	43.22%	22.24%	**0.001998**	0.021312
Bacteroides	Genus	42.83%	22.17%	**0.001998**	0.040245
Porphyromonadaceae	Family	3.74%	3.75%	0.993007	1
Parabacteroides	Genus	2.99%	2.91%	0.895105	1
Rikenellaceae	Family	2.09%	1.7%	0.462537	1
Alistipes	Genus	2.09%	1.7%	0.462537	1
Prevotellaceae	Family	14.88%	22.8%	0.208791	0.668131
Prevotella	Genus	11.08%	15.54%	0.348651	0.927543
Firmicutes	**Phylum**	20.45%	23.54%	0.167832	0.311688
Clostridia	Class	16.67%	19.23%	0.206793	0.475624
Clostridiales	Order	16.66%	19.2%	0.208791	0.584615
Clostridiaceae	Family	1.34%	0%	**0.030969**	0.198202
Lachnospiraceae	Family	4.01%	4.65%	0.381618	0.939367
Oscillospiraceae	Family	0%	1.42%	**0.041958**	0.223776
Negativicutes	Class	3.01%	2.28%	0.371628	0.610532
Ruminococcus	Genus	1.82%	2.27%	0.498501	1
Ruminococcaceae	Family	5.48%	5.58%	0.918082	1
Faecalibacterium	Genus	3.15%	2.46%	0.293706	0.898680
Selenomonadales	Order	1.08%	0%	0.519481	1
Selenomonadaceae	Family	1.06%	0%	0.519481	1
Megamonas	Genus	1.06%	0%	0.356643	0.931235
Acidaminococcales	Order	1.35%	0%	0.117882	0.380850
Acidaminococcaceae	Family	1.35%	0%	0.117882	0.443791
Phascolarctobacterium	Genus	1.35%	0%	0.110889	0.488605
Proteobacteria	**Phylum**	9.47%	10.17%	0.712288	0.841795
Betaproteobacteria	Class	4%	3%	0.090909	0.232323
Burkholderiales	Order	4%	3%	0.090909	0.318182
Sutterellaceae	Family	3.35%	2.62%	0.24975	0.729452
Sutterella	Genus	1.62%	1.13%	0.426573	1
Parasutterella	Genus	1.73%	0%	**0.003996**	0.070430
Gammaproteobacteria	Class	4.62%	6.27%	0.356643	0.610532
Enterobacterales	Order	4.23%	4.09%	0.927073	1
Enterobacteriaceae	Family	4.23%	4.09%	0.927073	1
Escherichia	Genus	2.94%	3.24%	0.824176	1
Aeromonadales	Order	0%	1.82%	**0.048951**	0.256993
Succinivibrionaceae	Family	0%	1.82%	**0.046953**	0.231153
Succinivibrio	Genus	0%	1.82%	**0.046953**	0.293967
Verrucomicrobia	**Phylum**	0%	1.45%	**0.000999**	0.002597
Spirochaetes	**Phylum**	0%	1.12%	0.077922	0.168831
Spirochaetia	Class	0%	1.12%	0.077922	0.232323
Spirochaetales	Order	0%	1.12%	0.077922	0.316592
Spirochaetaceae	Family	0%	1.12%	0.077922	0.331668
Treponema	Genus	0%	1.12%	0.077922	0.41736

The bold for values emphasizes the statistical significance.

### 3.5 Differences in the gut microbiome between Chinese and Africans

**Figure 3:**
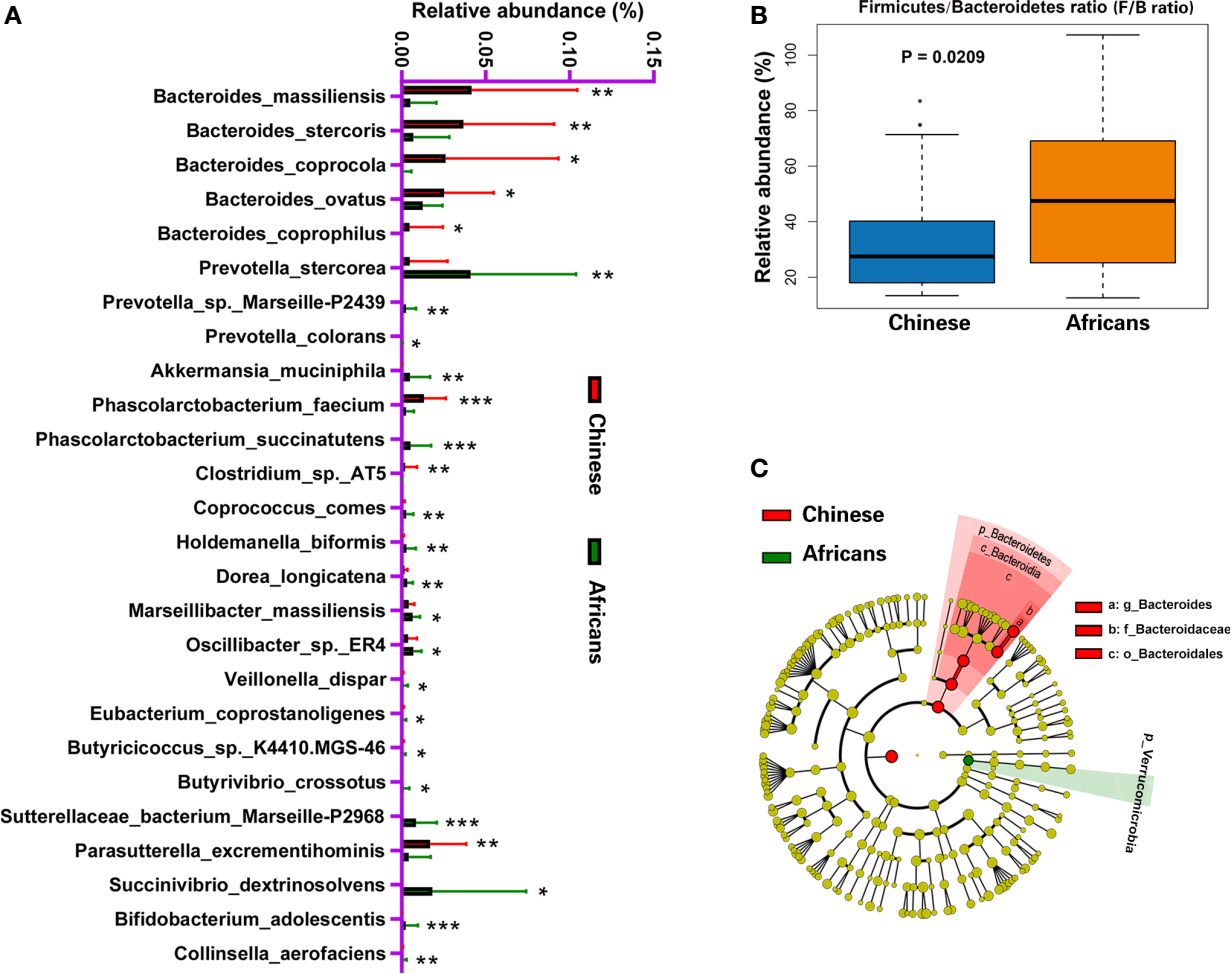
Distinct gut microbiota in Chinese and African groups. Bacterial species commonly and rarely present in either Chinese or Africans **(A)**. Boxplot showing the Firmicutes/Bacteroidetes ratio of community for the two groups **(B)**. The cladogram **(C)** was generated to depict the key and most differentially abundant taxa associated with ethnicity in Chinese (red) and Africans (green). Logarithmic LDA score = 3.8, and α-value = 0.05. *P < 0.05, **P < 0.01, ***P < 0.001.

Analysis of 16S rRNA sequence data using metastats revealed that 26 species, excluding the uncultured forms, differed significantly between the two groups (all, *p*<0.05, **Figure 3A**). Species such as *Bacteroides massiliensis*, *Bacteroides stercoris*, *Bacteroides coprocola*, *Bacteroides ovatus*, *Parasutterella excrementihominis*, *Phascolarctobacterium faecium*, *Bacteroides coprophilus*, and *Clostridium* sp. AT5 were significantly enriched in the Chinese group, whereas species such as *Akkermansia muciniphila*, *Prevotella colorans*, *Prevotella* sp. Marseille-P2439, *Prevotella stercorea*, *Phascolarctobacterium succinatutens*, *Succinivibrio dextrinosolvens*, *Sutterellaceae bacterium* Marseille-P2968, *Coprococcus comes*, *Holdemanella biformis*, *Dorea longicatena*, *Marseillibacter massiliensis*, *Oscillibacter* sp. ER4, *Veillonella dispar*, *Eubacterium coprostanoligenes*, *Butyricicoccus* sp. K4410.MGS-46, *Butyrivibrio crossotus*, *Bifidobacterium adolescentis*, and *Collinsella aerofaciens* were significantly enriched in the African group (all, *p*<0.05, **Figure 3A**). Wilcoxon rank-sum test revealed that the ratio of Firmicutes/Bacteroidetes increased significantly in the African group (*p* = 0.0209, **Figure 3B**). We further performed a metagenomic study based on linear discriminant analysis effect size (LEfSe) to identify the core taxa contributing to the differences between the two groups. The cladogram obtained from the LEfSe analysis showed that the Chinese group had a significant increase in the phylum Bacteroidetes, class Bacteroidia, order Bacteroidales, family Bacteroidaceae, and genus *Bacteroides*, whereas it had a significant decrease in the phylum Verrucomicrobia compared with the African group (**Figure 3C**).

### 3.6 Correlations between gut microbiota and parameters related to glucose metabolism

**Figure 4:**
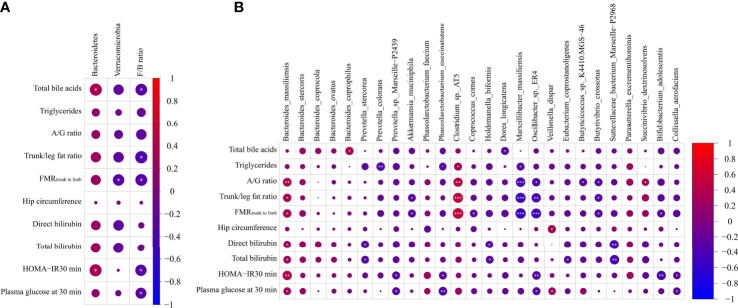
Heatmap spearman correlation analysis between parameters related to glucose metabolism and relative abundance of gut microbiome at both phylum **(A)** and species **(B)** levels in the Chinese group (n = 27) and African group (n = 29). *p < 0.05, **p < 0.01, ***p < 0.001.

To evaluate the correlation between gut microbiota and parameters related to glucose metabolism, we performed a Spearman correlation analysis of parameters related to glucose metabolism and microbiota abundance. In this regard, only data that showed significant differences were subjected to this analysis, and a heat map was used to depict these associations. The results demonstrated that the total bile acids positively correlated with phylum Bacteroidetes and species *B. coprophilus* and negatively associated with Firmicutes/Bacteroidetes ratio and *D. longicatena*. The levels of triglycerides positively correlated with *Clostridium* sp. AT5 and negatively correlated with *P. colorans*, *P. succinatutens*, and *M. massiliensis*. Measures of body fat distribution, A/G ratio, trunk/leg fat ratio, and FMR_trunk-to-limb_ positively correlated with *B. massiliensis* and *Clostridium* sp. AT5 and negatively associated with *M. massiliensis*, *Oscillibacter* sp. ER4 and *B. crossotus*. In addition, trunk/leg fat ratio and FMR_trunk-to-limb_ showed a negative association with Firmicutes/Bacteroidetes ratio and *A. muciniphila*, and FMR_trunk-to-limb_ also showed a negative association with phylum Verrucomicrobia and species *C. comes* and *B. adolescentis*, and A/G ratio was positively associated with *S. dextrinosolvens* and negatively correlated with *Butyricicoccus* sp. K4410.MGS-46. Another indicator of fat distribution, hip circumference, was positively associated with *V. dispar*. Total and direct bilirubin were positively associated with *B. massiliensis* and negatively correlated with *P. stercorea*, *H. biformis*, and *S. bacterium* Marseille-P2968, and total bilirubin was also correlated negatively with *E. coprostanoligenes*. Further associations were observed: lower levels of insulin sensitivity (HOMA-IR 30 min) and levels of plasma glucose at 30 min were positively associated with *B. massiliensis* and negatively correlated with Firmicutes/Bacteroidetes ratio, *Prevotella* sp. Marseille-P2439, *P. succinatutens*, *Oscillibacter* sp. ER4, and *Collinsella aerofaciens*; HOMA-IR 30 min was also positively correlated with phylum Bacteroidetes and negatively correlated with species *B. adolescentis*; and glucose 30 min positively correlated with species *V. dispar* (all *p* < 0.05, **Figures 4A, B**).

## 4 Discussion

This study is the first study to directly characterize the diversity and profile of gut microbiota in a group of adults and healthy Chinese and African subjects with NGT.

Accumulating studies have reported that gut microbiota acts as a crucial modulator of fat storage, glucose, and energy metabolism (12, 13). Currently, there is evidence that gut microbiota plays a causative role in the pathogenesis of metabolic disorders such as obesity, insulin resistance, and T2DM (10–12, 17, 18, 31), although many factors such as genetics and environment-related factors, including diets, lifestyle changes, geographical location, and migration, can shape the human gut microbiota community (19–25), leading to microbiota dysbiosis. This dysbiosis is associated with metabolic disorders (12, 13, 15, 16). Our study demonstrated host physiology–microbiota interactions in healthy individuals and also characterized specific bacteria associated with glucometabolic pathways. The metabolic disorders identified in our study included adiposity, hyperlipidemia, insulin resistance, and hyperglycemia. These metabolic disorders significantly increased as microbiota diversity, richness, and composition decreased in Chinese group with a body mass index (BMI) of 22.46 kg/m^2^ than in their African counterparts. These findings indicate that gut microbiota is associated with glucose regulation and utilization *in vivo*. The human microbiota is classified as the second genome due to its capability of carrying more than 98% of the genetic activity (32). Metagenomics is the study used to assess genetic material directly from environmental samples. In our study, we sequenced the V3–V4 region of the 16S rRNA gene to analyze the microbial community in the feces of Chinese and African groups. Rarefaction curves indicated that the diversity and abundance of gut microbiota in the Chinese group were relatively lower than those in the African group. Alpha diversity is a measure indicating various microbial species in stool samples. The higher Alpha diversity is an indicator of high abundance in a sample (33). In our study, the Alpha diversity measures included Chao 1 and observed species (indicator of microbiome richness), and Shannon index (indicator of microbiome richness and diversity). These indicators indicated lower microbiome richness and diversity in the Chinese group. Beta diversity is an indicator of gut microbiota heterogeneity between samples within each group. A higher beta diversity indicates greater differences in the composition of gut microbiota between samples in a specific group (33). We used the Bray–Curtis distance matrix to compare the heterogeneity in gut microbial communities and detected segregated clustering patterns in the Chinese and African groups, suggesting that the gut microbial community of the Chinese group is relatively unique from that of the African group. Collectively, these data indicate that the two groups were dissimilar to each other in the context of gut microbiota composition, richness, and diversity, despite the similar BMI. A high composition, high diversity, and microbiome stability are key indicators of healthy gut microbial communities (12). Recent findings demonstrate that a decline in the gut microbiota composition and diversity is linked with the prevalence of metabolic disorders (12). In both lean and obese individuals, low gut microbiome richness and diversity are linked with an increase in body adiposity, dyslipidemia, insulin resistance, and inflammation (34). Additionally, a defect in microbial diversity has been recently reported to reduce ecosystem functions and services (35). The human gut microbiota is a complex and diversified ecosystem with diverse bacteria that are dominated by the five major bacterial phyla, including Firmicutes, Bacteroidetes, Proteobacteria, Verrucomicrobia, and Actinobacteria (36). Among these, Firmicutes and Bacteroidetes account up to 90% of all gut bacteria (36). In contrast, our study detected that the top 5 phyla of the gut microbiota in adults were Bacteroidetes, Firmicutes, Proteobacteria, Verrucomicobia, and Spirochaetes. However, our study agrees with the aforementioned findings that Bacteroidetes and Firmicutes are the most prevalent in the microbiota. These differences may be attributed to the subject’s characteristics, various environmental factors, and genetic factors (21, 23), although high-throughput sequencing technology demonstrated that the most prevalent bacterial phyla are Bacteroidetes and Firmicutes both in the Chinese and African groups. However, their abundance differed between groups. Indeed, we observed several different species belonging to the Firmicutes phylum such as *P. succinatutens*, *C. comes*, *H. biformis*, *D. longicatena*, *M. massiliensis*, *Oscillibacter* sp. ER4, *V. dispar*, *E. coprostanoligenes*, *Butyricicoccus* sp. K4410.MGS-46, and *B. crossotus*, and species belonging to Bacteroidetes phylum such as *P. stercorea*, *Prevotella* sp. Marseille-P2439, and *P. colorans* were significantly abundant in the gut microbiome of the African group, whereas species belonging to phylum Firmicutes such as *P. faecium* and *Clostridium* sp. AT5 and species belonging to phylum Bacteroidetes such as *B. massiliensis*, *B. stercoris*, *B. coprocola*, *B. ovatus*, and *B. coprophilus* were enriched in the Chinese group. Both Firmicutes and Bacteroidetes are responsible for the metabolism of the carbohydrates (37). Firmicutes and Bacteroidetes are also involved in energy generation and conversion, transport and metabolism of amino acids, and production of short-chain fatty acids (SCFAs) (37). A great number of studies have extensively emphasized the contributions of microbiota to health and disease. In the gut, SCFAs hold a protective role against enteric/bacterial pathogens, thereby playing antimicrobial and anti-inflammatory activities (38). The SCFAs also augment energy expenditure and increase glucose tolerance by fostering gut motility and hormone secretion (39). We identified *Bacteroides* and *Prevotella* enterotypes whose functions are opposite. In humans, *Bacteroides* has been associated with high fat and protein intake (40), whereas *Prevotella* has been associated with high intake of fiber-rich diets (40). In addition, the genus *Prevotella* is known to produce great amounts of SCFAs (41), indicating the role of *Prevotella* in gut health status. Depletion in *Prevotella* may suggest a decrease in SCFAs, which are the major source of energy for enterocytes. This reduction in SCFAs increases mucosa permeability, resulting in bacterial translocation to the blood flow and extraintestinal organs (42) and, thus, metabolic disorders (43). Our results indicated that *Bacteroides* had significantly increased abundance in the Chinese group, while *Prevotella* had increased abundance in the African group. *Bacteroides* may become a member of the human flora immediately after birth, and species of this genus are genetic (44). *Bacteroides* is a genus that belongs to the phylum Bacteroidetes and has the capability to deconjugate and desiccate the primary bile acids and control their conversion into secondary bile acids (45). In addition, *Bacteroides* is known as an enterotoxin and can reduce insulin sensitivity by producing proinflammatory cytokines and lipopolysaccharides (4). These findings support our results regarding the effect of gut microbiota on host metabolism, and this could have resulted in several metabolic disorders observed, including excessive fat accumulation, hyperlipidemia, insulin resistance, and hyperglycemia. To date, *Bacteroides* is a recognized independent risk factor for T2DM (4). It is still completely unclear why Asians increase the incidence of diabetes at an early age for any given BMI than Africans and other ethnic groups, including Europeans (6, 7). It is noteworthy to mention that the Verrucomicrobia, a hallmark of glucose homeostasis and healthy gut, was more significantly prevalent in the African group only. This finding is of great importance considering these properties that are attributed to this microorganism, and these results represent a milestone baseline that will allow characterizing dysbiosis in the major diseases affecting the African population. *Akkermansia muciniphila*, a typical verrucomicrobia is the only detected species of this phylum in our study and enriched its abundance in favor of the African group. *Akkermansia muciniphila* is a Gram-negative, mucus-degrading bacterium with anti-inflammatory and immunostimulant functions and has probiotic properties (12). Previous studies have demonstrated that *A. muciniphila* can ameliorate obesity, insulin sensitivity, and endotoxinemia (46, 47). *Akkermansia muciniphila* can also regulate lipid metabolism, control adipocytes distribution, maintain glucose homeostasis, and restore gut barrier function (46, 47). *Akkermansia muciniphila* depletion is linked with obesity, insulin resistance, T2DM, and cardiometabolic disorders both in rodents and humans (11, 12), which suggests that the decline in this bacterium may also have significantly contributed to these metabolic disorders observed in our study. Accumulating evidence from animal studies shows that *A. muciniphila* can delay the onset of diabetes by promoting gut microbiota remodeling (48) and can also decrease fat mass development and alleviate dyslipidemia and insulin resistance (49). People with a higher abundance of *A. muciniphila* are characterized by a healthier metabolic status, especially in body fat distribution, triglycerides, and glucose levels, and have greater insulin sensitivity (46). This indicates that gut microbiome stability plays a prominent role in sustaining the host’s metabolic integrity, thereby contributing to energy harvest and metabolic regulation. It is surprising that Africans are more insulin resistant, while they have lower adiposity and good ability to secrete insulin (5, 8, 9). There is evidence suggesting the sex-specific pathways or responses to metabolic disorders, especially in Africans (50, 51). Indeed, our gender-specific analysis revealed that the African women were more likely to have aberrant glucose homeostasis, while men were more likely to have dyslipidemia that is characterized with abnormal LDL cholesterol. The present study has other important observation such as the detection of exclusive bacterial taxa in the Chinese and African groups. The relative abundances of Clostridiaceae and *Parasutterella* were significant and present in the Chinese group only, whereas the relative abundances of Aeromonadales, Oscillospiraceae, Succinivibrionaceae, and *Succinivibrio* were significantly in the African group and absent in Chinese group, suggesting the distinct microbiota signatures associated with these groups. Moreover, our study found phylum Spirochaetes that was previously reported in hunter–gatherer populations to be enriched in the African group and absent in the Chinese group (21). The presence and role of these taxa in the gut microbiota of Chinese and Africans should be examined in more detailed large-scale studies to confirm the present findings. The gut microbiota regulates various host metabolic pathways, which physiologically link the gut, pancreas, liver, adipose tissue, skeletal muscle, and brain *via* multiple mechanisms, including (1) energy extraction by absorption and digestion of monosaccharides and fibers into SCFAs, (2) modulation of fat storage *via* SCFAs, and (3) translocation of bacteria and their products by binding to G-protein-coupled receptors (GPCRs) that are expressed by enteroendocrine cells (12). The gut-microbiota-mediated pathways further interacted with the production of gut hormones such as GLP-1, leading to enhanced energy expenditure, decreased food intake, and improved lipid and glucose metabolism and insulin biosynthesis (secretion) and sensitivity (12). Gut microbiota also affects host metabolism by modulating various metabolites including bile acids (52). Bile acids are important signaling molecules and act as metabolic regulators that support digestion by facilitating intestinal absorption and transport of lipids (53). Excessive accumulation of bile acids in the liver or circulation results in malabsorption of fat and deposition of toxic xenobiotics and endobiotics, and this can damage cells and organs in the gastrointestinal tract (53). Gut dysbiosis is the term commonly used to refer to unbalanced gut microbiota, which is associated with an unhealthy outcome (54). Dysbiosis of the gut microbiota leads to improper microbial-derived metabolite signaling, intestinal barrier dysfunction, oxidative stress, and immune dysregulation (12). This dysbiosis can also cause abnormal aryl hydrocarbon receptor (AHR) and GLP-1 resistance and decrease G-protein receptor expression, which result in the development of obesity, insulin resistance, and T2DM (Zhang, 12, 13, 43, 55). The Firmicutes/Bacteroidetes ratio (F/B ratio) has been proposed as an important marker for gut microbial dysbiosis (56). A recent study comparing insulin-sensitive and insulin-resistant obese subjects found that the Firmicutes/Bacteroidetes ratio increased as insulin sensitivity increased (57), suggesting its role in glucose–insulin homeostasis. Our study found that the Chinese group had a significant decrease in Firmicutes content and Firmicutes/Bacteroidetes ratio compared with African group. The change in (F/B ratio) is associated with various metabolic disorders in humans (56, 58) and has negative correlations with glucose levels (58), which is in agreement with our study findings. Further correlations between gut microbiota and factors that are involved in glucose metabolism were observed. For example, we found that the phylum Bacteroidetes was positively correlated with total bile acids and HOMA_-IR30min_, and the species *B. massiliensis* belonging to Bacteroidetes was positively associated with A/G ratio, trunk/leg fat ratio, FMR_trunk-to-limb_, HOMA_-IR30min_, and levels of bilirubin and glucose (glucose 30 min). Verrucomicrobia was negatively correlated with FMR_trunk-to-limb_, and its species *A. muciniphila* was negatively associated with both trunk/leg fat ratio and FMR_trunk-to-limb_. These results indicate that the gut microbiome composition may be implicated in the modulation of glucose metabolism in non-obese conditions. Our study combined both data from the two groups to analyze associations between the gut microbiome and parameters of glucose metabolism; this should be regarded as a confounding factor. An individual’s genetic makeup affects the composition of the key microbiome (20). For example, the microbiota of identical twins living separately is significantly more alike than those of uncoupled individuals (20). Contrarily, the environment appears to have minor significance, since married couples did not have a significantly higher similarity of microbial communities than uncoupled individuals, even though these couples lived in the same environment with similar dietary practices (19). In the same manner, in a 16S rRNA sequencing study comparing the gut microbiota of 2,084 subjects from many different countries who live in the same city, the genetic background explained the dissimilarities in microbiome composition (23). However, a recent study by Vengay and collaborators investigated the impact of migration on microbiome composition and showed that migration to the United States profoundly affects the microbiome in the long term even after several generations (24), indicating that migrancy has an important impact on health. Overall, our results showed that there were associations between the gut microbiota, host physiology, and glucometabolic pathways, which can play a significant role in the occurrence and evolution of metabolic disorders. Despite the importance of understanding the connection between environmental factors, host genetics, microbiota, and health disparities, there are no findings on how the baseline gut microbiotas of Chinese and African healthy individuals are linked with their metabolic phenotypes. We observed differences in the gut microbiome that were associated with the metabolic phenotypes of the two groups. Although the microbiome status at the group level is very different, there were some overlaps, too. These differences in microbiome composition may be explained by the factors such as genetic background, current diet and lifestyle, and even more by migrancy. The present study revealed diverse gut microbiome and metabolic phenotypes in two closely matched healthy groups of people who have lived in the same city for at least a year and characterized specific microbiome associated with glucometabolic pathways. Our LEfSe and metastats analyses found differentially abundant and core bacterial taxa in the Chinese and African groups, and these taxa could be potential biomarkers. This study has some limitations. We did not collect information about the lifestyle and dietary nutrition of participants to evaluate if there was any association between nutrient intake or physical activity level (lifestyle) and differences in the composition of the gut microbiota. Another limitation is that the sample size was too small and included only healthy subjects. Further large-scale longitudinal studies wherein the subjects are followed over a long period of time (evaluation from insulin sensitive to obesity to insulin resistance and T2DM) would confirm a potential and dynamic change in microbiome status, genetic diversity, and general metabolic response with diverse statuses of glucose metabolism, and could determine causality.

## 5 Conclusion

This study gives evidence of an interaction between the gut microbiome, host physiology, and glucometabolic pathways, and this could contribute to adiposity and pathophysiology of dyslipidemia, insulin resistance, and hyperglycemia. Interestingly, the gut microbiota reveals a high abundance of the phylum Bacteroidetes in the Chinese group and phylum Verrucomicrobia and Firmicutes/Bacteroidetes ratio (F/B ratio) in the African group. Furthermore, the abundance of some bacteria related to metabolism was associated with glucose-metabolism-related parameters. These findings provide an important basis for determining the relation between the gut microbiome and the pathogenesis of various metabolic disorders and constitute the road map to examine further mechanisms related to gut dysbiosis in the disease conditions.

## Data availability statement

The data presented in this study are deposited in the NCBI repository, accession number PRJNA853567.

## Ethics statement

This study was reviewed and approved by Medical Ethics Committee of Xiangya Hospital of Central South University. The patients/participants provided their written informed consent to participate in this study.

## Author contributions

MXL and PN conceived this study. PN, MXL, and BYX established the analysis design. MXL, PN, XHL, CJL, and ZHL contributed to the statistical analysis plan. PN, BYX, LRL, LPL, TTL, and MJ were responsible for data collection and statistical analyses. PN wrote the manuscript. All authors contributed to the interpretation of the findings and the manuscript’s critical revision. All authors have read and approved the final version of the manuscript. MXL and PN had full access to all the data in the study and take responsibility for the integrity of the data and the accuracy of the data analysis.

## Funding

This project was supported by the National Natural Science Foundation of China (No. 81170753) and the Natural Science Foundation of Hunan Province (No. 2015SK20302). The study sponsor/funder was not involved in the design of the study and in the collection, analysis, and interpretation of data or writing of the report and did not impose any restrictions regarding the publication of the report.

## Acknowledgments

We thank all study participants for their cooperation, and we also thank the Staff in Endocrinology Clinical Laboratory for their excellent technical assistance. We gratefully acknowledge the guidance of Professors Dongmei Zhang and Lijuan Guo in the Department of Endocrinology, Xiangya Hospital of Central South University.

## Conflict of interest

The authors declare that the research was conducted in the absence of any commercial or financial relationships that could be construed as a potential conflict of interest.

Data are presented as percentage (%), and were calculated between the relative abundance of bacterial taxa at multiple taxonomic levels.

## Publisher’s note

All claims expressed in this article are solely those of the authors and do not necessarily represent those of their affiliated organizations, or those of the publisher, the editors and the reviewers. Any product that may be evaluated in this article, or claim that may be made by its manufacturer, is not guaranteed or endorsed by the publisher.
