# Supplementary figures and images for: Gut microbiota is associated with differential metabolic characteristics: A study on a defined cohort of Africans and Chinese
Source: Front Endocrinol (Lausanne). 2022 Sep 28;13:942383. doi: 10.3389/fendo.2022.942383 (PMC9554505; doi:10.3389/fendo.2022.942383)

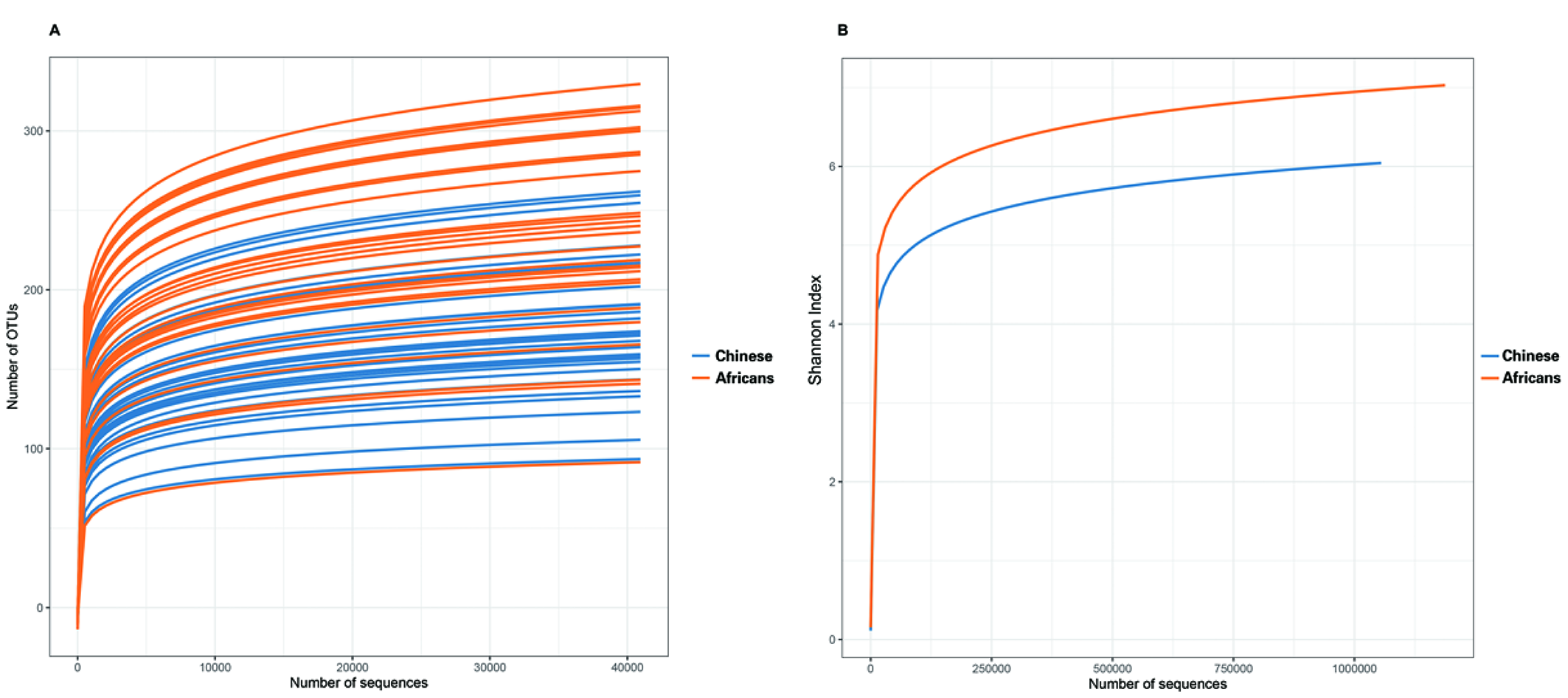

Supplement: Supplementary file 1 [file Image_1.tif]

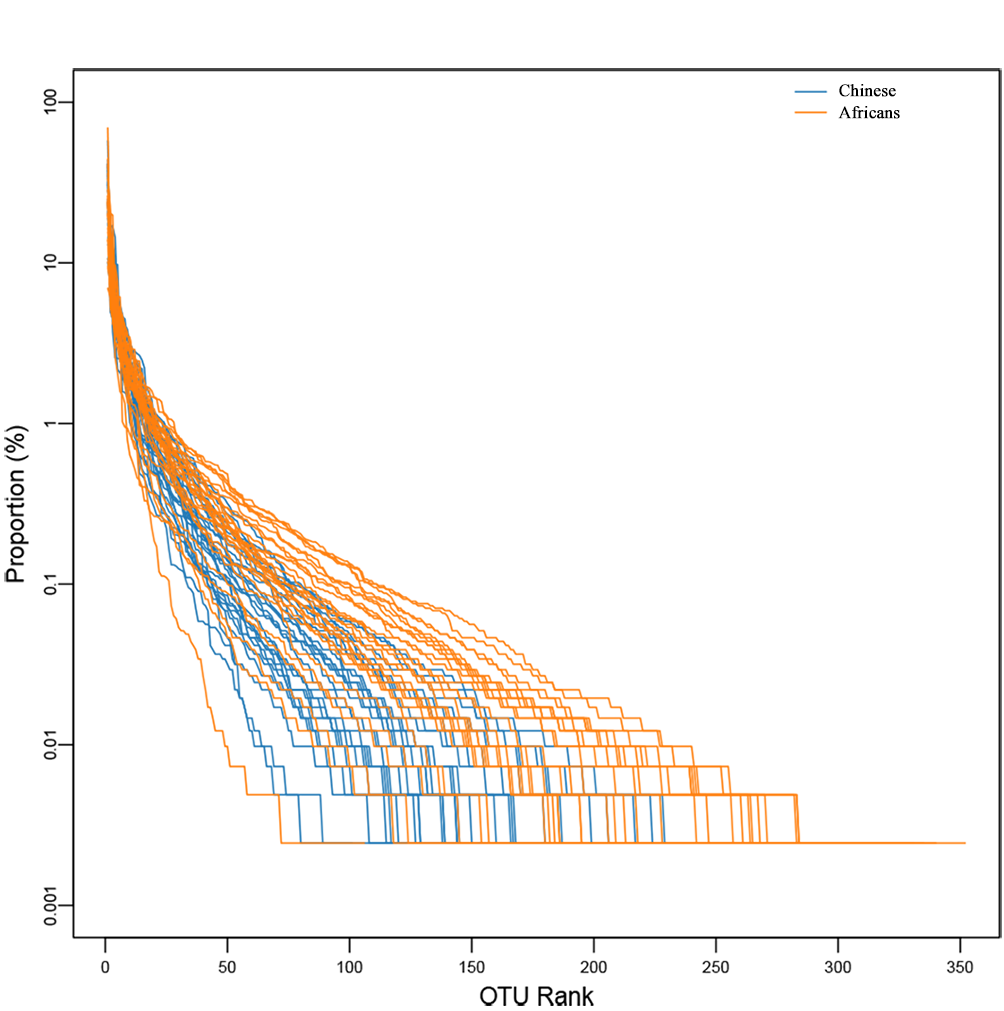

Supplement: Supplementary file 2 [file Image_2.tif]
