# Supplementary material for: Gut microbiota is associated with differential metabolic characteristics: A study on a defined cohort of Africans and Chinese
Source: Front Endocrinol (Lausanne). 2022 Sep 28;13:942383. doi: 10.3389/fendo.2022.942383 (PMC9554505; doi:10.3389/fendo.2022.942383)
Supplement: Supplementary file 3 [file DataSheet_1.docx]

Supplementary Material

# Supplementary Data

The rarefaction curves (**Supplementary Figure 1A**) and Shannon diversity curves (**Supplementary Figure 1B**) were computed to reflect the diversity of samples and evaluate whether the sequencing data of samples are reasonable. In addition, the rank-abundance curves were generated to depict relative species abundance, species richness, and evenness (**Supplementary Figure 2**).

# Supplementary Figures and Tables

**Supplementary Table S1. Data from OGTT at each time point**

| **Characteristics** | **Chinese (n=27)** | **Africans (n=29)** | **P value** |
| --- | --- | --- | --- |
| Plasma glucose (mmol/L) |  |  |  |
| Fasting | 4.58±0.40 | 4.61±0.46 | 0.774 |
| 30 min | 7.26±1.22 | 6.55±1.30 | **0.041** |
| 60 min | 6.20±1.59 | 6.24±1.68 | 0.941 |
| 120 min | 5.04±0.91 | 5.21±1.10 | 0.532 |
| 180 min | 3.96±0.83 | 4.14±1.05 | 0.466 |
| Plasma insulin (µU/mL) |  |  |  |
| Fasting | 6.97±2.25 | 6.56±2.42 | 0.513 |
| 30 min | 62.37±21.45 | 54.66±29.06 | 0.115 |
| 60 min | 64.76±38.83 | 52.44±24.99 | 0.129 |
| 120 min | 37.66±25.47 | 33.22±14.93 | 0.617 |
| 180 min | 11.58±9.78 | 12.93±10.13 | 0.533 |
| Plasma GLP-1 (ng/mL) |  |  |  |
| Fasting | 0.28±0.33 | 0.19±0.08 | 0.652 |
| 30 min | 2.53±1.91 | 1.98±1.46 | 0.176 |
| 60 min | 2.53±2.23 | 1.84±1.06 | 0.283 |
| 120 min | 1.67±1.91 | 1.08±0.64 | 0.594 |
| 180 min | 0.48±0.56 | 0.40±0.32 | 0.850 |

Data are (X̅ ± SD)

**Supplementary Table 2. Analysis of participants’ characteristics based on gender**

| **Characteristics** | **Male** | | **P.value** | **Female** | | **P.Value** | |
| --- | --- | --- | --- | --- | --- | --- | --- |
|  | **Chinese** | **Africans** |  | **Chinese** | **Africans** |  | |
|  | **(n=19)** | **(n=21)** |  | **(n = 8)** | **(n = 8)** |  | |
| Age (y) | 25.00 ± 1.73 | 26.95 ± 4.11 | 0.318 | 25.25 ± 0.71 | 23.88 ± 3.98 | 0.097 | |
| Height (cm) | 174.60 ± 5.92 | 176.30 ± 6.08 | 0.375 | 164.63 ± 5.83 | 164.63 ± 6.89 | >0.999 | |
| Weight (kg) | 70.79 ± 7.83 | 70.52 ±10.34 | 0.928 | 55.75 ± 4.10 | 59.38 ± 11.10 | 0.401 | |
| BMI (kg/m^2^) | 23.22 ± 2.26 | 22.73 ± 3.30 | 0.586 | 20.63 ± 2.08 | 22.00 ± 4.38 | 0.439 | |
| Waist circumference (cm) | 78.62 ± 11.16 | 80.90 ± 8.71 | 0.472 | 70. 00 ± 6.78 | 76.38 ± 10.07 | 0.245 | |
| Hip circumference (cm) | 91.38 ± 10.06 | 97.04 ± 8.75 | 0.065 | 88.94 ± 6.53 | 92.63 ± 10.38 | 0.409 | |
| Waist-hip ratio | 0.86 ± 0.07 | 0.83 ± 0.04 | 0.159 | 0.79 ± 0.04 | 0.82 ± 0.03 | 0.083 | |
| Arm circumference(cm) | 29.11 ± 3.50 | 28.27 ± 3.21 | 0.431 | 24.41 ± 1.90 | 24.80 ± 2.77 | 0.618 | |
| Systolic BP (mmHg) | 116.2 ± 7.30 | 115.8 ± 9.09 | 0.879 | 104.50 ± 9.35 | 102.38 ± 7.07 | 0.616 | |
| Diastolic BP (mmHg) | 74.68 ± 8.47 | 70.38 ± 5.88 | 0.068 | 69.13 ± 8.17 | 68.13 ± 7.55 | 0.803 | |
| SMI (kg/m^2^) | 16.40 ± 1.71 | 16.10 ± 1.91 | 0.600 | 13.41 ± 1.16 | 12.75 ± 1.63 | 0.365 | |
| LS-BMD (g/cm^2^) | 0.97 ± 0.08 | 1.02 ± 0.11 | 0.112 | 0.99 ± 0.11 | 1.00 ± 0.11 | 0.915 | |
| PF-BMD (g/cm^2^) | 0.99 ± 0.13 | 1.02 ± 0.15 | 0.518 | 0.95 ± 0.12 | 0.93 ± 0.11 | 0.660 | |
| Total body BMD (g/cm^2^) | 1.16 ± 0.05 | 1.17 ± 0.09 | 0.835 | 1.18 ± 0.08 | 1.10 ± 0.07 | **0.043** | |
| Body fat (%) | 25.03 ± 5.28 | 23.49 ± 6.53 | 0.420 | 30.89 ± 4.01 | 36.4 ± 7.17 | 0.079 | |
| A/G ratio | 1.17 ± 0.13 | 0.92 ± 0.17 | **<0.0001** | 0.83 ± 0.05 | 0.77 ± 0.15 | 0.329 | |
| FMRtrunk-to-limb | 1.19 ± 0.19 | 0.89 ± 0.17 | **<0.0001** | 0.86 ± 0.12 | 0.76 ± 0.13 | 0.126 | |
| Trunk/leg fat ratio | 1.12 ± 0.20 | 0.90 ± 0.14 | **0.000** | 0.81 ± 0.08 | 0.80 ± 0.09 | 0.733 | |
| Total cholesterol (mmol/L) | 4.12 ± 0.54 | 4.33 ± 0.98 | 0.413 | 4.47 ± 0.99 | 4.46 ± 0.55 | 0.980 | |
| LDL cholesterol (mmol/L) | 1.37 ± 0.29 | 2.51 ± 0.89 | **<0.0001** | 2.54 ± 0.99 | 2.60± 0.58 | 0.882 | |
| HDL cholesterol (mmol/L) | 1.37 ± 0.29 | 1.53 ± 0.36 | 0.123 | 1.61 ± 0.17 | 1.54 ± 0.32 | 0.567 | |
| Triglycerides (mmol/L) | 1.07 ± 0.66 | 0.96 ± 0.56 | 0.291 | 0.93 ± 0.39 | 0.52 ± 0.15 | **0.021** | |
| Total bile acids (µmol/L) | 5.36 ± 5.67 | 3.60 ± 3.27 | 0.180 | 5.39 ± 5.66 | 2.30 ± 1.50 | 0.161 | |
| Total bilirubin (µmol/L) | 14.87 ± 5.72 | 12.10 ± 5.60 | 0.130 | 13.98 ± 4.52 | 9.70 ± 5.14 | 0.099 | |
| Direct bilirubin (µmol/L) | 6.57 ± 2.41 | 5.21 ± 2.42 | 0.084 | 6.46 ± 2.01 | 4.05 ± 1.80 | **0.024** | |
| Fasting GLP-1 (ng/mL) | 0.33 ± 0.39 | 0.19 ± 0.09 | 0.236 | 0.17 ± 0.11 | 0.18 ± 0.03 | 0.065 | |
| Fasting insulin (µU/mL) | 7.27 ±2.36 | 6.65 ± 2.71 | 0.447 | 6.28 ± 1.90 | 6.34 ± 1.55 | 0.944 | |
| Fasting glucose (mmol/L) | 4.68 ± 0.38 | 4.57 ± 0.49 | 0.430 | 4.34 ± 0.35 | 4.72 ± 0.34 | **0.040** | |
| Δglucose(30-0min) | 2.71 ± 1.12 | 1.92 ± 1.26 | **0.044** | 2.62 ± 1.03 | 2.01 ± 0.99 | 0.242 | |
| DI_180_ | 87.91 ± 27.49 | 103.5 ± 37.82 | 0.390 | 114.7 ± 24.01 | 139.6 ± 46.36 | 0.199 |  |
| Matsuda index | 6.99 ± 2.40 | 8.97 ± 4.56 | 0.151 | 7.30 ± 2.80 | 6.75 ± 1.90 | 0.655 |  |
| HOMA-IR30min | 21.19 ± 9.96 | 16.16 ± 12.01 | **0.037** | 18.38 ± 4.13 | 16.89 ± 6.35 | 0.721 | |

Data are (X̅ ± SD)

**Supplementary Table 3. Sequencing reads data of this study**

| Sample ID | Number of Reads (R1+R2) | Total V3-V4 Sequences | Quality Score  (Q30) |
| --- | --- | --- | --- |
| 1 | 91214 (96.29%) | 80314 (84.78%) | 97.1% |
|  |  |  |  |
| 2 | 82090 (96.59%) | 72873 (85.75%) | 97.12% |
| 3 | 118352 (96.73%) | 105484 (86.21%) | 97.16% |
| 4 | 73921 (95.85%) | 64806 (84.03%) | 96.95% |
| 5 | 83077 (96.53%) | 73510 (85.41%) | 97.12% |
| 6 | 77258 (95.06%) | 68560 (84.36%) | 97.14% |
| 7 | 81007 (96.09%) | 71241 (84.5%) | 96.94% |
| 8 | 86301 (96.5%) | 75818 (84.78%) | 96.92% |
| 9 | 95008 (96.12%) | 84051 (85.03%) | 97.14% |
| 10 | 74122 (95.22%) | 65350 (83.96%) | 97.05% |
| 11 | 84035 (96.87%) | 74791 (86.21%) | 97.15% |
| 12 | 96473 (96.45%) | 85413 (85.4%) | 97.16% |
| 13 | 93277 (95.71%) | 81419 (83.54%) | 96.84% |
| 14 | 104423 (96.67%) | 92616 (85.74%) | 97.13% |
| 15 | 105078 (96.3%) | 92855 (85.1%) | 97.17% |
| 16 | 97221 (95.9%) | 85585 (84.42%) | 97.02% |
| 17 | 98525 (96.54%) | 86926 (85.18%) | 97.09% |
| 18 | 96454 (95.29%) | 83606 (82.59%) | 96.58% |
| 19 | 77855 (94.9%) | 68824 (83.89%) | 97.08% |
| 20 | 92095 (96.33%) | 81229 (84.96%) | 97.01% |
| 21 | 83134 (95.96%) | 73165 (84.45%) | 96.98% |
| 22 | 84710 (96.12%) | 74725 (84.79%) | 97.09% |
| 23 | 101403 (96.35%) | 89439 (84.98%) | 97.03% |
| 24 | 109221 (96.43%) | 96249 (84.97%) | 96.97% |
| 25 | 111903 (96.03%) | 97800 (83.92%) | 96.89% |
| 26 | 94475 (96.2%) | 83323 (84.84%) | 96.99% |
| 27 | 93667 (96.2%) | 82185 (84.41%) | 96.93% |
| 28 | 86244 (96.3%) | 75613 (84.43%) | 96.94% |
| 29 | 79394 (95.98%) | 69591 (84.13%) | 96.91% |
| 30 | 83695 (96.26%) | 73250 (84.25%) | 96.78% |
| 31 | 62076 (96.43%) | 54950 (85.36%) | 96.97% |
| 32 | 92269 (96.06%) | 80632 (83.95%) | 96.85% |
| 33 | 78985 (95.86%) | 68938 (83.67%) | 96.81% |
| 34 | 104382 (95.88%) | 91167 (83.74%) | 96.81% |
| 35 | 118916 (96.47%) | 104875 (85.08%) | 97.06% |
| 36 | 93337 (96.05%) | 81601 (83.97%) | 96.89% |
| 37 | 104808 (96.89%) | 92997 (85.97%) | 97.11% |
| 38 | 95499 (96.89%) | 84884 (86.12%) | 97.04% |
| 39 | 100782 (96.3%) | 89076 (85.12%) | 97.07% |
| 40 | 94135 (95.97%) | 82063 (83.66%) | 96.74% |
| 41 | 88334 (96.28%) | 77581 (84.56%) | 96.95% |
| 42 | 91604 (96.22%) | 80455 (84.51%) | 96.97% |
| 43 | 101691 (96.56%) | 90164 (85.62%) | 97.09% |
| 44 | 72047 (96.3%) | 63595 (85%) | 97.02% |
| 45 | 64565 (95.05%) | 56550 (83.25%) | 96.82% |
| 46 | 98306 (95.78%) | 85609 (83.41%) | 96.82% |
| 47 | 63851 (94.58%) | 56275 (83.36%) | 96.96% |
| 48 | 90540 (96.15%) | 79690 (84.63%) | 97.05% |
| 49 | 73673 (96.49%) | 65384 (85.64%) | 97.16% |
| 50 | 99871 (96.03%) | 87713 (84.34%) | 96.96% |
| 51 | 101033 (95.98%) | 88412 (83.99%) | 96.85% |
| 52 | 89008 (96.21%) | 78761 (85.14%) | 97.06% |
| 53 | 71492 (96.23%) | 62808 (84.54%) | 96.88% |
| 54 | 108317 (96.29%) | 95150 (84.58%) | 96.91% |
| 55 | 108169 (96.44%) | 95227 (84.9%) | 97.01% |
| 56 | 75075 (94.75%) | 65339 (82.46%) | 96.79% |

**Supplementary Table 4. Unmapped reads in the two groups**

| Taxon | Chinese (n = 27) | Africans (n=29) | P value | FDR |
| --- | --- | --- | --- | --- |
| Phylum | 1511 (3.69%) | 3726 (9.10%) | **0.000999** | 0.002597 |
|  |  |  |  |  |
| Class | 1651 (4.03%) | 5028 (12.28%) | **0.000999** | 0.007659 |
|  |  |  |  |  |
| Order | 1793 (4.38%) | 5077 (12.40%) | **0.000999** | 0.013986 |
| Family | 3481 (8.50%) | 8210 (20.05%) | **0.000999** | 0.021312 |
| Genus | 5975 (14.59%) | 12823 (31.32%) | **0.000999** | 0.040245 |
| Species | 10776 (15.06%) | 16942 (26.32%) | **0.000999** | 0.020626 |
|  |  |  |  |  |

## Supplementary Figures

##
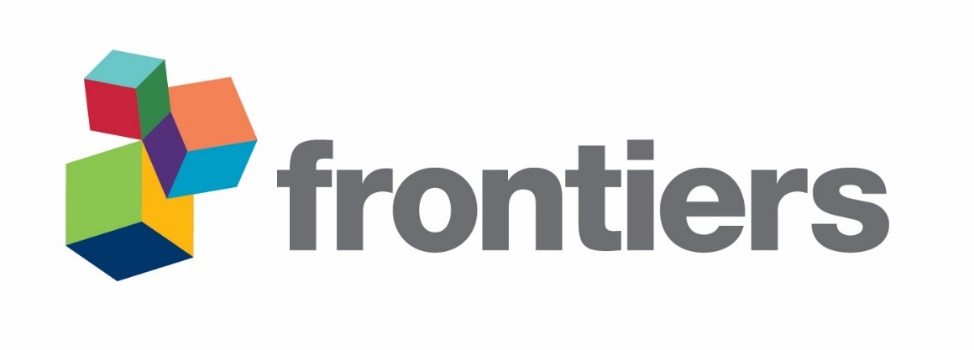


**Supplementary Figure 1. Feasibility Analysis and Pyrosequencing Reads of All Samples From the Two Groups**. Rarefaction curves (A) and Shannon diversity index curves (B) for OTUs were computed. Each curve represents a sample, and different colors represent OTUs associated with groups.

**Supplementary Figure 2. Rank-abundance curves.**
